# Sexsomnia - a detailed approach to evaluation

**DOI:** 10.3389/frsle.2026.1847000

**Published:** 2026-06-11

**Authors:** Colin M. Shapiro, Persis F. Yousef, Julian A. Gojer, Naheed K. Hossain, Chris Y. Kim

**Affiliations:** 1 Sleep on the Bay, Toronto, ON, Canada; 2 Department of Psychiatry and Ophthalmology, University of Toronto, Toronto, ON, Canada; 3 University Health Network (UHN), Toronto, ON, Canada; 4 Horizon Health Network, Fredericton, NB, Canada; 5 Royal Ottawa Health Care Group, Ottawa, ON, Canada

**Keywords:** forensic psychiatry, NREM parasomnia, NREM sleep, obstructive sleep apnea (OSA), parasomnia, sexsomnia, sexsomnia patient assessment, sleep-related sexual behavior

## Abstract

Sexsomnia is an underrecognized parasomnia with significant clinical, interpersonal and legal implications. This article proposes a comprehensive, structured framework for the evaluation of sleep-related sexual behaviors, aimed at improving diagnostic clarity and risk assessment. Rather than relying on isolated features, the approach emphasizes a multidimensional assessment across multiple domains, recognizing that no single factor is determinative and that not all indicators carry equal diagnostic weight. An indexed framework of 19 domains is presented, covering presenting complaints, levels of awareness during events, collateral reports, sleep history, environmental and physiological triggers, medical, psychiatric and family history, medication and substance use, lifestyle factors and prior investigations. Additional domains address behavioral indicators of risk, relationship and social context, safety planning and legal and forensic considerations. Overlapping features across domains are acknowledged, with emphasis placed on patterns across categories rather than cumulative scoring. This structured approach is intended to assist clinicians in identifying features that increase the likelihood of sexsomnia, distinguishing it from other conditions and addressing associated safety and legal concerns. The framework aims to support consistent clinical evaluation, facilitate interdisciplinary communication and inform risk management in both clinical and forensic contexts.

## Preamble

Public awareness of sexsomnia has expanded over time, including its depiction in an episode of the television series, *House, M.D*. Parallel to this, the condition has gained increasing recognition within academic literature as a distinct clinical entity since its earlier description nearly two decades ago in the *Canadian Journal of Psychiatry* by Shapiro et al. (2003). Reflecting this shift, a 2021 review by Holoyda et al. (2021) in the *Journal of the American Academy of Psychiatry and the Law* noted that approximately half of the 27 cited articles explicitly included the term “sexsomnia” in their titles.

Sexsomnia is generally classified as a subtype of non-rapid eye movement (NREM) parasomnias, a group of arousal disorders that also includes sleepwalking, sleep talking and bruxism. According to the *Diagnostic and Statistical Manual of Mental Disorders, Fifth Edition, Text Revision* (American Psychiatric Association, 2022), these conditions involve partial arousals from deep sleep accompanied by complex behaviours performed without full awareness (see criteria below).

Despite this clinical framework, sexsomnia continues to be underrecognized in practice, even though epidemiological findings suggest it may be more common than previously thought, with lifetime prevalence estimates ranging from 7.1% to 10.1% and a 6.1% “recent” prevalence defined as over the past three months (Pallesen et al., 2025). It is worth noting that these values are based on self-reported survey data.

###  Criteria

A. Recurrent episodes of incomplete awakening from sleep, usually occurring during the first third of the major sleep episode
B. No or little dream imagery is recalled
C. Amnesia for the episode is present
D. Clinically significant distress or impairment
E. Disturbance is not attributable to the effects of a substance
F. Coexisting mental or medical conditions do not explain the episodes
G. Diagnosed with NREM sleep arousal disorder, sleepwalking type, accompanied by sleep-related sexual behaviour (sexsomnia).

The treatment is described as including the following (American Psychiatric Association, 2022):

1. Sleep hygiene
2. Avoidance of alcohol and other drugs
3. Optimizing the sleep environment
4. Patient education about sexsomnia and the risk it poses to potential sleeping partners
5. Clonazepam is the medication treatment of choice for NREM parasomnias, including sexsomnia

The report describes a number of legal cases some of which were viewed as bizarre with several described as “penetrative sexual behaviours involving the defendant's fingers or genitalia” (Holoyda et al., 2021). It is notable that the article includes the statement that: “The defendant was acquitted in all eight cases in which the verdict was known.”

There are other series which have not had the same overwhelming result. This was more likely in cases where there was not a formal sleep study, which can show features of parasomnia. One way of understanding this is the dominant view that there are many forms of parasomnia including: talking in sleep; walking in sleep (somnambulism); singing in sleep; laughing in sleep (hypnogely); eating in sleep; inappropriate voiding (urine) in sleep and driving in sleep.

The recent review notes “sexsomnia has traditionally been thought to rise from a primary sleep disorder, rather than a paraphilic disorder or other underlying sexual disorder” (Holoyda et al., 2021). We, the authors of this article, agree with that. It is almost certain that the majority of cases of sexsomnia occur in the context of an ongoing relationship and although somewhat unusual, is often tolerated by the spouse. However, it can lead to significant marital friction, especially if in unusual situations, e.g., two lawyers sharing a bed with a young child after flying from Ireland to Australia. The mother - who is aware that her husband occasionally has intercourse with her and she is sure that he is asleep, but when she wakes up to see her husband (who she realizes is asleep) performing oral sex on their two-year-old who is sharing the bed (atypically) with a couple. This ends in a divorce.

Holoyda et al. (2021) (4 physicians from the United States and one from New Zealand) provides a table headed: “Conditional release considerations for patients with sexsomnia”, which include the following:

1. Avoidance of triggers
2. Abstinence from alcohol and other drugs
3. Avoiding shift work that impacts on sleep stress reduction measures
4. Risk-reduction strategies
5. Treatment with clonazepam
6. Treatment of other sleep disorders
7. Not sleeping with bed partner
8. Sleeping with the door locked to prevent sleepwalking

The paper concludes that with the growing recognition of sexsomnia as a legitimate NREM parasomnic disorder, the diagnosis is likely to be invoked with increasing frequency in legal proceedings both by individuals with a bona fide sleep disorder and by those seeking to avoid criminal culpability.

## 1 Introduction

This article attempts to provide a comprehensive approach to evaluating sexual behaviors in sleep. The index below lists the factors that may be of relevance to episodes of sexsomnia. No patient will have all of these and the approach should be there if they are a number of items in a number of categories, there is an increased likelihood of sexsomnia. There is an overlap of some items in more than one category. A superscript “o.l.” has been added to alert the readers that this item occurs elsewhere. Repeated “points” do not add extra weight (validity), but to facilitate their diagnosis, there will typically be some items from several sections. Not all items have equal valence and some items overlap with others in different sections. We advise that this paper may contain explicit sexual content that may be considered graphic in nature and warrants reader discretion. The subject pertains to an area of medico-legal importance in which explicit issues must necessarily be addressed. Such explicit descriptions, as encountered by the authors in clinical practice, may assist clinicians in identifying relevant features when examining patients with suspected sexsomnia.

The following provides an “Index” of sections:

- Presenting complaints
- Awareness and consciousness during events
- Bed partner concerns/observer report
- Sleep history
- Sleep environment triggers
- Factors that contribute to disruption of deep sleep
- Factors that contribute to arousals
- Medical and neurological history
- Psychiatric history
- Medications and substance use
- Family history including epilepsy
- Indicia of sexsomnia
- Behaviors and indicia (signs) of risk pertinent to sexsomnia
- Relationship, social and legal context relevant to sexsomnia
- Investigation process
- Risk and safety assessment
- Sleep study and previous evaluations
- Lifestyle factors
- Legal and forensic considerations

## 2 Subsections and discussion

### 2.1 Presenting complaints

a. Describe the episodes: what sexual behaviors occur (e.g., masturbation, pelvic thrusting, fondling a partner, central vocalizations and intercourse attempts)
b. Duration: how long does the episode last before resolution?
c. First onset: when did the behavior begin?
d. Frequency: how often do the episodes occur? (nights per week or per month)
e. Pattern: do the episodes increase or decrease in frequency?

The patient presents with recurrent episodes of inappropriate or atypical sexual behaviors arising from sleep, consistent with a sleep-related parasomnia. Reported behaviors may include masturbation, pelvic thrusting, attempts at intercourse, sexual touching of a bed partner or sexual vocalizations, occurring without clear conscious intent. Episodes typically last from several seconds to a few minutes and often resolve spontaneously or after external interruption (Schenck et al., 2007; American Academy of Sleep Medicine, 2023). Initial onset is commonly reported weeks to years prior to clinical presentation, frequently beginning in adolescence or early adulthood. The frequency of episodes varies, ranging from isolated monthly events to multiple episodes per week, often occurring during the first half of the night when slow-wave sleep (SWS) predominates. Patterns over time may show increased frequency during periods of sleep deprivation, stress, alcohol use or irregular sleep schedules, with potential reduction following improved sleep hygiene or targeted treatment (Schenck et al., 2007; Howell, 2012; American Academy of Sleep Medicine, 2023).

### 2.2 Awareness and consciousness during events

a. Any recollection of the episodes?
b. Confusional disorientation upon waking?
c. The enactment episode(s) appears to be more “dreamy, blank, frequent, vivid, complex or violent”
d. Amnesia of the episode

Patients generally report partial or complete amnesia for the episodes of sexsomnia (Shapiro et al., 2003). When recollection is present, it is usually fragmentary and poorly organized rather than a clear narrative memory. Patients may exhibit transient confusion, grogginess or disorientation upon awakening, consistent with incomplete arousal from NREM sleep. Many individuals describe the episodes as “blank,” without associated dream imagery, while others report vague dream-like sensations without explicit sexual content. Volitional control and conscious awareness during the events are typically absent, supporting the characterization of these behaviors as automatic rather than intentional (Mahowald and Schenck, 2005; Pressman, 2007; American Academy of Sleep Medicine, 2023).

### 2.3 Bed partner/observer reports

a. What has the partner witnessed?
b. Are the behaviors aggressive, consensual or non-responsive?
c. Any injury to the partner or patient/accused?
d. Does the accuser report difficulty “waking” the patient?
e. Are there observed breathing patterns e.g., snoring or restless movements before the episode?

Bed partners frequently describe the patient initiating sexual behaviors abruptly while appearing asleep, often with eyes closed or with a fixed, non-responsive facial expression (Shapiro et al., 2003; Holoyda et al., 2021). One partner reported that her husband would often say, “that is enough of that,” then roll over and continue sleeping when she believed he may have been asleep during intercourse. The behaviors are commonly described as automatic and non-interactive, with limited or absent responsiveness to verbal redirection. While overt aggression is uncommon, behaviors may be intrusive or distressing. Accidental physical or emotional harm to the partner has been reported in some cases. Partners often note difficulty waking the patient during episodes. Observers may also report preceding features such as restless movements, snoring, irregular breathing or partial arousals prior to the onset of sexual behaviors, suggesting sleep instability as a contributing factor (Schenck et al., 2007; Pressman, 2007; Zadra et al., 2008).

### 2.4 Sleep history

a. Bedtime routine, sleep duration and sleep deprivation
b. Insomnia (difficulty in initiating/maintaining sleep)
c. Nightmares, night terrors and dream enactment
d. History of other parasomnias (e.g., sleepwalking, sleep-talking, teeth grinding, etc)^°.*l*.^
e. Sleep paralysis or hallucinations
f. Excessive daytime sleepiness
g. Sleep deprivation/interrupted sleep

A detailed sleep history frequently reveals irregular sleep schedules, insufficient total sleep time or chronic sleep deprivation, all of which are recognized precipitants of NREM parasomnias (American Academy of Sleep Medicine, 2023). Patients may report insomnia symptoms, particularly difficulty maintaining sleep with frequent nocturnal awakenings. There is often a personal or childhood history of other parasomnias, including sleepwalking, sleep talking or confusional arousals. Some individuals also report nightmares or dream enactment behaviors, raising the need for careful differential diagnosis. Episodes of sleep paralysis or hypnagogic hallucinations may be present but are less common. Excessive daytime sleepiness is frequently reported, reflecting disrupted or non-restorative sleep and reinforcing the role of sleep instability in the pathophysiology of these events (Mahowald and Schenck, 2005; Howell, 2012; Stallman and Kohler, 2016).

### 2.5 Sleep environmental triggers

a. Sleeping in unfamiliar places
b. Sleeping next to different partners
c. Room temperature, noise and interruptions
d. Use of phone, alcohol or stimulants before bed

Environmental and behavioral factors may also contribute to sleep fragmentation and arousal instability. Sleeping in unfamiliar environments, sharing a bed with different partners and exposure to suboptimal bedroom conditions, such as inappropriate room temperature, environmental noise or frequent interruptions, can disrupt normal sleep architecture (Fietze et al., 2016; Hu et al., 2024). In addition, pre-sleep behaviors including mobile phone use and the consumption of alcohol or stimulants may further disrupt sleep continuity (Riha et al., 2023). Together, these factors increase the likelihood of nocturnal arousals and may act as precipitating conditions for parasomnias and sexsomnia in susceptible individuals.

### 2.6 Factors that contribute to disruption of deep sleep

a. Poor sleep hygiene/routine^°.*l*.^
b. Noisy environment^°.*l*.^
c. Ambient temperature and cool body temperature fluctuations
d. Lack of normal melatonin and/or magnesium levels
e. Heavy exercise
f. Excessive caffeine intake

Multiple behavioral, environmental and physiological factors can contribute to the disruption of SWS. Poor sleep hygiene and irregular sleep routines undermine sleep continuity, while exposure to a noisy environment can increase nocturnal arousals and sleep fragmentation (Fietze et al., 2016; Hu et al., 2024). Ambient temperature and fluctuations in core body temperature may further disrupt the maintenance of deep sleep (Harding et al., 2019). In addition, deficiencies or dysregulation of sleep-related neurobiological factors, such as melatonin and magnesium, may adversely affect sleep depth and stability (Arab et al., 2023). Heavy exercise close to bedtime and excessive caffeine consumption are also well-recognized contributors to reduced SWS by increasing physiological arousal and delaying sleep onset (Korkutata et al., 2025).

### 2.7 Factors that contribute to arousals

a. Positive family history of arousals
b. Rotating or night shift work
c. Other sleep disorders (including hypersomnia and insomnia)
d. Insufficient sleep
e. Uncontrolled stress
f. Psychiatric disorders, such as bipolar and depressive disorders^°.*l*.^
g. Sleep deprivation^°.*l*.^.
h. Periodic limb movement disorder (PLMD)
Alcohol consumption/withdrawal^°.*l*.^
j. Sleep-disordered breathing
k. Psychotropic medications or side effects of other medications
l. Drugs of abuse^°.*l*.^
m. Disrupted circadian rhythms (circadian rhythm disorders)
n. Forced awakening from sleep
o. Physical contact with bed partner/mate

A wide range of pathophysiological, psychological and environmental factors have been associated with arousals from sleep (Mainieri et al., 2021). These include a positive family history of arousal disorders: rotating or night shift work, co-occurring sleep disorders such as circadian rhythm disorders, hypersomnia, insomnia, PLMD and sleep apnea and insufficient sleep or sleep deprivation. Additional contributors include uncontrolled stress, bipolar and depressive disorders, alcohol use or withdrawal, the use of psychotropic medications or adverse effects of other medications and the use of drugs of abuse. Disruptions to circadian rhythms, forced awakenings from sleep and physical contact with a bed partner have also been identified as potential precipitating factors (Mainieri et al., 2021). Collectively, these factors can increase sleep instability and promote partial arousals, which may precipitate episodes of sexsomnia.

### 2.8 Medical and neurological history

a. History of head injury/concussion or seizures
b. Symptoms of obstructive sleep apnea: snoring, gasping for air and witnessed apneas^°.*l*.^
c. Restless legs syndrome or periodic limb movements in sleep (RLS/PLMS)^°.*l*.^
d. Chronic illnesses (e.g., diabetes, thyroid disorders)
e. Any neurological disorders (Parkinson's disease, epilepsy)
f. History of sleep deprivation and interrupted sleep^°.*l*.^
g. Amnesiac episode^°.*l*.^

As with all cases of sleep-related sexual activity, a comprehensive medical history is required as part of the general work-up. General medical disorders that impair sleep quality may exacerbate a pre-existing parasomnia or precipitate one (Schlarb et al., 2021). A history of blackouts, seizures or cardiovascular diseases that could precipitate changes in sleep or pre-existing sleep disorders should be addressed (Iranzo, 2018). Endocrine disorders involving hormonal dysfunction can disrupt sleep and conversely, sleep disorders can impair hormonal regulation. Of note, diabetes, thyroid disorders and adrenal dysfunction may affect sleep in many ways, including poor sleep quality, confusional states and parasomnias (Iranzo, 2018).

Adult-onset parasomnias, including REM behavior disorder and confusional arousals, which may be associated with sleep-related sexual behaviors, should prompt evaluation for underlying neurologic conditions such as early-onset dementia, Parkinson's disease, seizure disorders, tumors/malignancies and multiple sclerosis (Iranzo, 2018).

### 2.9 Psychiatric history

a. History of psychiatric disorder (e.g., depression, anxiety)^°.*l*.^
b. Post-traumatic stress disorder (PTSD; especially sexual trauma)
c. Substance use disorder^°.*l*.^
d. History of dissociative symptoms (mental processes involving a disconnection between a person's thoughts, memories, feelings, actions or sense of identity)
e. Psychological trauma
f. Impulse control disorders
g. Negative emotions (e.g., guilt)
h. Conflicted sexuality
High level of stress^°.*l*.^

Sleep history is often part of a psychiatric history and is addressed elsewhere in this paper. However, assessment for major mental disorders, including schizophrenia and bipolar mood disorders, is important not only for their clinical management but also for evaluation of the reliability of the patient's history, as symptoms such as bizarre thoughts and behaviors, mania, hypersexuality and impaired or inconsistent memory for sleep-related behaviors may be present (Sprecher et al., 2015).

Dissociative disorders may present as sexsomnia (Cankardas and Schenck, 2020). However, in the absence of a documented history of dissociative experiences, such a diagnosis may have limited evidentiary value in legal proceedings. Dissociative disorders may theoretically occur in either the accused or the alleged victim. One of the authors (JG) had a case in which the complainant alleged being in a dissociative state during sexual activity with the accused. The proceedings were discontinued partway through the trial for various reasons and no determination of guilt was reached.

Disturbances in sleep and neurocognitive function, as described in schizophrenia by Sprecher et al. (2015), may have broader relevance to psychiatric conditions in which cognition, mood regulation and executive functioning are affected. In this context, disorders such as bipolar disorder and major depressive disorder may also influence attention, memory and affective stability, which can in turn be relevant to the consistency and reliability of patient testimony in forensic settings.

### 2.10 Medication and substance use

a. Selective serotonin reuptake inhibitors/serotonin norepinephrine reuptake inhibitors (SSRIs/SNRIs)
b. Tricyclics (TCAs)
c. Antipsychotics
d. Sedative-hypnotics (e.g., zolpidem, zopiclone)
e. Dopamine agonists.
f. Benzodiazepines
g. Cannabis
h. Alcohol (major trigger)
Recreational drugs (e.g., cocaine, 3,4-methylenedioxymethamphetamine or MDMA)^°.*l*.^
j. Caffeine and energy drinks^°.*l*.^

Medications such as tricyclic antidepressants (TCAs), selective serotonin reuptake inhibitors (SSRIs) and serotonin norepinephrine reuptake inhibitors (SNRIs) and benzodiazepines can trigger or worsen parasomnias (Kierlin and Littner, 2011). Other drugs implicated in parasomnic activity are beta blockers, zolpidem and zopiclone, topiramate, montelukast and lithium (Howell, 2012).

Both alcohol and stimulants can disrupt sleep, trigger hypersexual behaviors and induce psychotic states that influence sexual activity (Munro, 2020; Holoyda et al., 2021). Psychedelics, including psilocybin mushrooms, lysergic acid diethylamide (LSD) and *N, N*-dimethyltryptamine (DMT), can precipitate toxic or drug-induced psychosis, confusion and altered mental states that may explain sleep-related sexual behaviors (Jiménez-Correa et al., 2022; Sabé et al., 2025). These effects must be distinguished from primary parasomnia, such as sexsomnia. Frequently, multiple substances may be consumed concurrently and determining the contribution of each drug, as well as the effects of their combination, may require consultation with a toxicologist.

The role of alcohol in sleep-related sexual behaviors is complex. Alcohol is known to disrupt sleep and may contribute to sleep-related sexual acts (Munro, 2020). As a behavioral disinhibitor, it can also lead to impulsive sexual behaviors (Cartwright, 2014; Munro, 2020). In cases of extreme intoxication, automatic behaviors may occur, which must be distinguished from alcohol-related blackouts.

The role of alcohol in criminal liability for alleged unwanted sexual behaviors during sleep is nuanced and must be carefully contextualized. While some classifying systems may exclude a diagnosis of sexsomnia in the presence of alcohol, a thorough analysis of the circumstances, including the degree of intoxication and associated typical behaviors, is essential, taking into account all relevant medical and legal considerations.

Careful reasoning that the behaviors are secondary to a parasomnia, rather than resulting from extreme intoxication, may provide a viable defense depending on the legal jurisdiction. In contrast, memory loss due to a blackout does not constitute a defense and the possibility of malingering must also be thoroughly ruled out.

If the use of drugs, alcohol or their combinations has the potential to trigger a parasomnia, several legal hurdles need to be overcome before concluding that sexsomnia occurred in a specific case. In jurisdictions where any defense is barred if a violent offense is associated with drug or alcohol use, culpability may be resolved at the outset. However, if the substance caused the individual to lose voluntary control, the distinction between sexsomnia and other automatism becomes less relevant, as the behavior is considered involuntary. In such jurisdictions that recognize drug-induced automatisms, a defense may be available.

### 2.11 Family history

a. Family history of parasomnia (e.g., somnambulism)
b. Family history of epilepsy^°.*l*.^
c. Any psychiatric disorders in close relatives

Evidence suggests that a family history of certain conditions may increase vulnerability to parasmonia (Battiato et al., 2025) and possibly sexsomnia. Individuals with first-degree relatives who have experienced parasomnias, such as sleepwalking or sleep-related paraphilias, appear to be at higher risk, indicating a potential genetic or familial predisposition (Battiato et al., 2025). Similarly, a family history of epilepsy has been associated with increased susceptibility to sleep disorders involving abnormal arousals (Manni and Terzaghi, 2010). Additionally, the presence of psychiatric disorders in close relatives may further enhance the risk, likely reflecting shared genetic, neurobiological or environmental factors that contribute to sleep instability and vulnerability to parasomnia (Ollila et al., 2024).

### 2.12 Indicia of sexsomnia

a. Previous history of parasomnia^°.*l*.^
b. Family history of parasomnia^°.*l*.^
c. Reasons to have increased SWS^°.*l*.^:

i. Increase physical activity
ii. Sleep deprivation
iii. Late onset of sleep
iv. Alcohol consumption (especially in the first half of the night) or medications

d. Psychological stress^°.*l*.^
e. Negative emotions associated with relationship: anger, confusion, denial, frustration, guilt, revulsion and shame
f. Sleep disruption secondary to obstructive sleep apnea, noise for young children and jet lag
g. Physical proximity (a safe sleep environment and potentially sleeping arrangements for those with parasomnias, such as sleepwalking)^°.*l*.^
h. Sleep-related epilepsy (e.g., pelvic thrusting, sexual arousal, orgasm)
i. Certain medication (e.g., zolpidem)^°.*l*.^
j. Amnesia of the episodes^°.*l*.^

The information above highlights that multiple factors must be considered when determining whether an individual exhibits features of sexsomnia. In the 23 years since the term was first introduced, there has been broad recognition that some individuals engage in sexual behaviors during sleep (Shapiro et al., 2003). When such behaviors occur within a well-established couple, they may be unusual but generally do not result in legal consequences. However, when they occur between relative strangers, they can lead to significant legal implications. In this section, we present illustrative case examples that are relevant to the medico-legal context.

If the sexual interaction is not consensual and the protagonist is awake, this is clearly a version of non-consensual sexual contact. However, if the active partner is asleep, most courts would generally consider that the individual was not acting voluntarily and therefore is not legally responsible for their actions. A key concern in such cases is whether the active party might be “feigning sleep” to avoid a charge of sexual assault.

There are many features that may lead to the greater likelihood that the active party was asleep at the time of the physical contact. Invariably, there is no third-party to opine on the subject and an assessment needs to be made, based on the circumstances.

Three adults (two men, one woman) shared accommodations during a joint holiday. The sleeping arrangement involved two beds pushed together and light social contact occurred before sleep. During the night, the female participant awoke distressed, reporting that one of the male participants engaged in sexualized contact with her while she was asleep. Initially, she believed he was asleep, but she subsequently interpreted the event as non-consensual sexual contact occurring during her sleep. Different jurisdictions view the process of a person having an action, but not being aware of it as grounds for an acquittal. There are some restrictions that view this as “a disease of the mind” which has many ramifications. The unwelcomed closeness invariably leads to adverse consequences. There is also the possibility of feigning sleep.

A prepubescent child experienced a parasomnia episode during the night. The child awoke and went to the bathroom, then proceeded to the kitchen to prepare food, still holding a utensil. During this episode, the child entered the bed of another child who was asleep and, under the misperception of a threat, unintentionally caused severe injury to the other child. These actions are presumed to have occurred while the child was not fully conscious. This case illustrates the potential for serious harm during complex behaviors associated with parasomnia.

In many long-term relationships, sexual behavior occurring while one partner is asleep may be perceived as consensual, tolerated or occasionally welcomed. One clinical case involved an adult male patient who was a law enforcement officer and had been acquitted of driving under the influence of alcohol following a high-profile sporting event. As part of routine clinical follow-up, he was seen regularly by the clinician. During these sessions, it was noted that he engaged in sexual behavior during sleep. The patient reported awareness of such behavior only upon discussion in the clinical setting and his partner noted that these behaviors were sometimes more pronounced during sleep than while awake, which she generally perceived as non-problematic within their relationship.

This case illustrates that sexual behavior during sleep can occur in familiar partnerships without resulting in conflict or perceived harm. In contrast, similar behavior involving individuals who are not familiar to the person exhibiting the behavior is typically considered non-consensual and may be classified as assault. Sleep-related sexual behavior, such as other complex parasomnias (e.g., sleepwalking, sleep-talking or sleep-driving), is generally regarded as non-volitional. Most jurisdictions do not consider such behavior criminal if the individual is not consciously aware of their actions. However, legal interpretations vary and some jurisdictions may adopt a stricter approach.

### 2.13 Behaviors and indicia (signs) of risk pertinent to sexsomnia

a. Standard indicia of sexsomnia
b. Previous history of parasomnia^°.*l*.^
c. Family history of parasomnia^°.*l*.^
d. Reasons to have increased SWS^°.*l*.^:

i. Increased physical activity
ii. Sleep deprivation
iii. Late onset of sleep
iv. Alcohol consumption (especially in the first half of the night)
v. Sedating medications

e. Psychological stress^°.*l*.^
f. Negative emotions associated with relationships: anger, confusion, denial, frustration, guilt, revulsion and shame^°.*l*.^
g. Sleep disruption associated with relationships
h. Physical proximity
i. Noise (e.g., young children)

A number of psychosocial and behavioral risk factors pertinent to sexual behavior during sleep have been identified in the past two decades. Riha et al. (2023) recently conducted an analysis of 335 consecutive patients who presented with NREM parasomnias from 2005 through 2020 to a single sleep center in the United Kingdom (UK) to identify features distinguishing sexsomnia from other parasomnias. The study compared patients with sexualized sleep behaviors to a control group of 270 patients with confirmed NREM parasomnias without sexual behaviors in sleep.

In that study, of the sixty-five patients identified with confirmed features of sexual behavior during sleep, 58 were male and seven were female, with the mean age at presentation of 33 years ± 9.5 years (Riha et al., 2023). The main reason for referral for all 65 patients (including three homosexual patients) was engaging in sexual behavior during sleep consistent with their sexual orientation. Twenty-two patients (19 males and three females; 53.7%) reported that their sexual behavior during sleep occurred more than once per week, whereas 16 patients (16 males and no females; 39%) reported the episodes occurring only a few times per month. The onset of sexual behavior during sleep was more frequent in adulthood, while that of NREM parasomnia of the control group was more common in childhood (61.1% vs. 52.9%; *p* = 0.007). None of the 65 patients found the episodes “pleasurable”, reporting them instead as “distressing”, particularly in relation to the detrimental impact on bed partners directly affected. It was unclear whether the individual who committed the act found the episode distressing based on their own recollection or if the distress arose upon learning about it from their partner.

#### 2.13.1 Potential precipitating factors for sexual behaviors in sleep

In the study by Riha et al. (2023), seventeen patients (30.4%) reported that their sexual behavior during sleep was exacerbated by alcohol consumption, of unspecified alcohol types, content and amounts, which was significantly more compared to the control group (*p* < 0.001). Relationship difficulties were identified as a trigger by six patients with sexsomnia (10.7%), compared to the control group (eight patients; 3%). Stress was reported as a trigger by 30 of 56 patients (53.6%). Twelve patients (21.4%) reported that sleep deprivation exacerbated their sexual behavior during sleep, with increases in both the frequency and intensity of these behaviors. Sleep deprivation or extreme fatigue may be considered a trigger, as it increases “sleep pressure” and destabilizes deep sleep. However, other predisposing factors may be more influential as many healthy individuals experience sleep deprivation without developing sexsomnia. Seven of 56 patients were working in the armed forces or the police. Patients with sexsomnia were significantly more likely to report co-occurring sleepwalking or as previously evinced sleepwalking. Males typically attended clinic with their bed partner, while females usually presented alone (Riha et al., 2023). It remains uncertain whether the prevalence and features of sexual behaviors during sleep vary across different cultures or socioeconomic groups.

### 2.14 Relationship, social and legal context relevant to sexsomnia

- Alcohol and drug use^°.*l*.^
- Impact on relationship or partner distress
- Issues with consent, guilt, embarrassment
- Fear of sleeping next to someone
- Bed sharing versus sleeping alone
- Legal or social consequences as a result of sexsomnia

Sexsomnia often has major interpersonal, clinical and occasional criminal consequences. The disorder is typically chronic and may lead to significant relationship and marital strain, including accusations of sexual assault or rape within marriage and can result in legal charges (Ingravallo et al., 2014). Sexsomnia may negatively affect the sexual health of initiators as well as contribute to adverse relationship and emotional outcomes (Mangan, 2004; Trajanovic et al., 2007). Klein and Houlihan (2010) reported that individuals with sexsomnia experienced lower levels of relationship and sexual satisfaction and higher levels of sexual problems compared with healthy controls (Klein and Houlihan, 2010).

A web-based survey by Mangan in 2004 categorized experiences associated with sleep-related sexual behavior, including psychosocial difficulties affecting both initiators and recipients of sleep sex (Mangan, 2004). Among 121 respondents (mean age 37 years; 65% female), six primary problem areas were identified: fear and lack of emotional intimacy; guilt and confusion; feelings of repulsion and sexual abandonment; shame, disappointment and frustration; annoyance and suspicion and embarrassment and a sense of self- incrimination.

In our 2006 study, data were obtained over a 3-month period via a 28-item internet-based survey assessing sexual behavior during sleep (Trajanovic et al., 2007). The sample comprised 219 respondents (69% male; mean age 30 years, range 15 years −67 years), of whom 92% reported experiencing multiple episodes of sexsomnia. Sexual intercourse during sleep was reported by 48% of participants. Identified precipitating factors included physical contact with another person in bed (64%), stress (52%), fatigue (41%), alcohol use (14.6%) and drug use (4.3%). Sexsomnia involving minors during sleep was reported by 5.9% of respondents (10 males, three females), all of whom reported legal repercussions. Nearly half of the sample (47%) also reported having another diagnosed sleep disorder.

Dubessy et al. (2017) reported in a case series of 17 patients, predominantly women, that sexsomnia was frequently associated with marital difficulties, including feelings of strangeness, guilt, shame and depression. One case reported significant psychological distress characterized by suicidal ideation (Dubessy et al., 2017; Toscanini et al., 2021). Dubessy and colleagues also described a 76-year-old married woman in this French series who experienced recurrent episodes of sleep-related masturbation accompanied by sexual vocalizations (Dubessy et al., 2017). During these episodes, she repeatedly called out the name of a man who was not her husband and denied knowing anyone by that name (Dubessy et al., 2017). Sleep talking is non-volitional or a conscious choice and does not reflect waking mentation. Therefore, it is not admissible as evidence in legal proceedings (Bornemann et al., 2006) and should not be interpreted as conscious behavior or as indicative of “subconscious” motivation in clinical contexts. Sleep talking during the sexsomnic act needs to be explored in greater detail in terms of nature of the vocalizations, complexity of what is said and relevance to the behavior in question. In addition, Riha et al. (2023) reported that 11 of 56 patients with sleep-related sexual behavior identified in their study (21.6%) experienced problematic relationships, compared with 27 control participants (10%).

Seeking professional help for sexsomnia can be challenging and several factors influence help-seeking behavior. First, the presence of a bed partner is often necessary for the condition to be recognized. Without a partner, individuals with sexsomnia may remain unaware of the behavior due to the inherent amnesia associated with the disorder. Second, a problem is identified in cases where either there is a bed partner who suffers in some way or the “sexsomniac” repeatedly hurts oneself physically from vigorous sleep masturbation. Moreover, there is often considerable embarrassment involving sexsomnia, which prevents people from talking to a physician or other healthcare professionals about it (Guilleminault et al., 2002). Some individuals may not recognize sleep disorders including sexsomnia as a “medical condition”, instead attributing it to psychological or personal issues and therefore may not seek professional help (Rauch et al., 2024).

Sexsomnia can have significant legal implications. In a 2019 review of 351 forensic referrals to a sleep center, 41% (*n* = 145) were related to sexual assault allegations. Of these initial referrals, 31% (*n* = 110) were considered potentially sleep-related and were accepted for forensic sleep evaluation (Cramer Bornemann et al., 2019). In that study, cases were rejected if the behavior was better explained by another medical or psychiatric condition or if there was concomitant alcohol intoxication or illicit drug use. Among the 110 cases accepted for evaluation, 52 involved sexual assault allegations. Sexsomnia was the most frequent diagnosis, accounting for 46 of the 110 cases, followed by disorders of arousal (*n* = 22), pharmaceutical toxicity related to zolpidem or zaleplon (*n* = 18) and sleep deprivation (*n* = 7). Notably, none of the 110 referrals were diagnosed as malingering.

### 2.15 Investigation process of a case of sexual activity when the individual claims to be asleep

a. Review the records of the charge and victim witness statements
b. Interview the client taking an exhaustive psychiatric and medical history
c. Arrange for a thorough physical examination, along with a full by chemical and psychological screen
d. Obtain past medical and psychiatric reports
e. Arrange for an electroencephalogram and brain scanning if indicated by history and physical exam
f. Interview relatives of the client
g. Obtain the victim's account of the events
h. Arrange for PSG
Consider phallometric testing i.e. erotic preference
j. Consider a blood or urine screen for drug abuse
k. Psychological testing with a special emphasis on personality testing
l. Psychodiagnostics looking at all DSM-V axes
m. Evaluate psychopathy
n. Generate a differential diagnosis and rule out any malingering
o. Consider obtaining a second opinion from an independent expert without conflicts of interest

**Figure 1:**
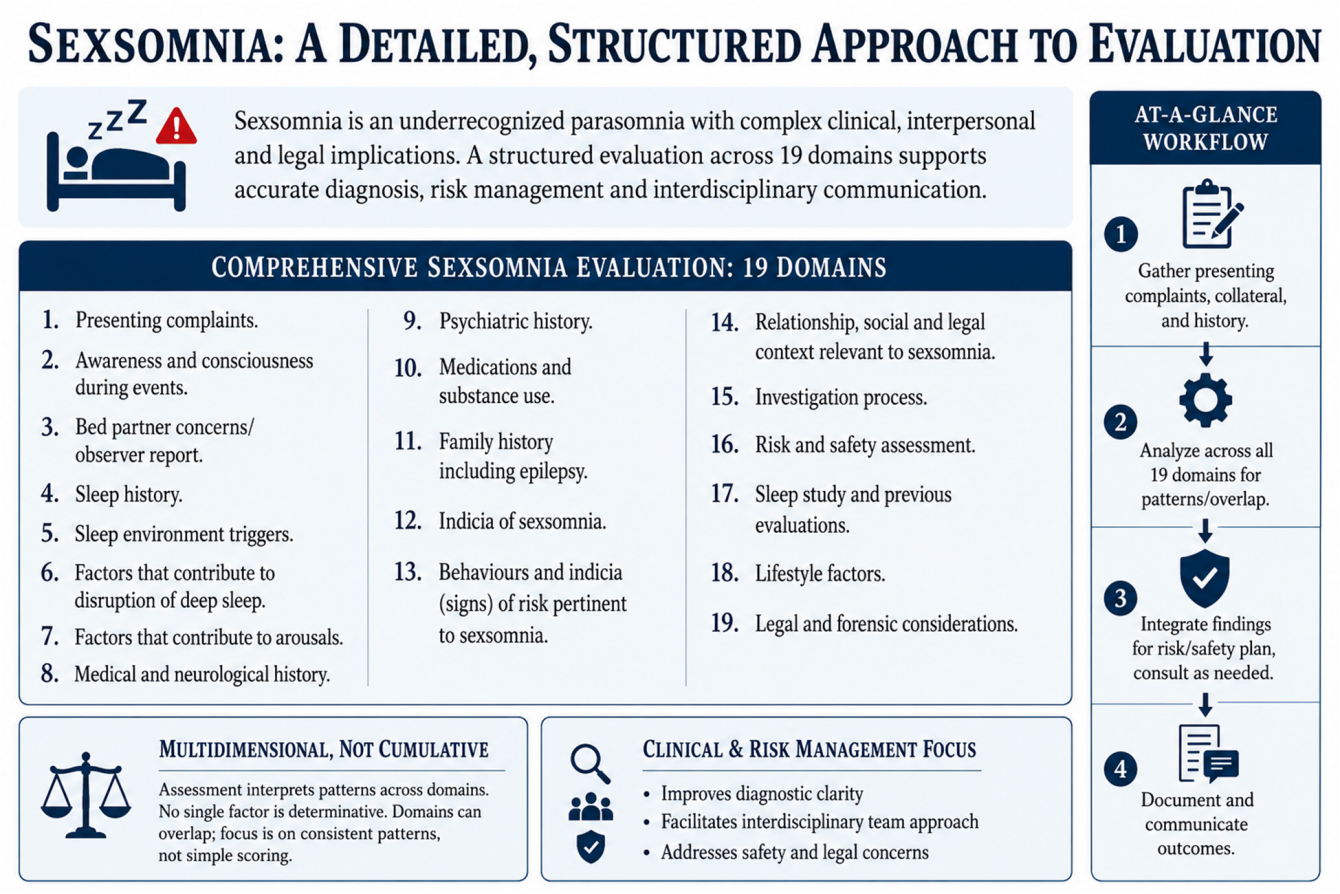
Infographic illustrating a structured approach to the evaluation of sexsomnia. The framework encompasses nineteen comprehensive assessment domains, including presenting complaints, sleep history, psychiatric history, family history, legal considerations and risk assessment. A four-step workflow guides clinicians through the processes of collecting patient-reported concerns, analyzing relevant domains, integrating clinical findings and documenting outcomes. The model emphasizes the multidimensional and overlapping nature of assessment, as well as the importance of multidisciplinary risk management, to support accurate diagnosis and address potential legal and safety concerns.

Investigation of an alleged sexsomnia episode, particularly in a forensic context, typically requires a multidisciplinary approach (See Figure 1). Appendix Table 1 outlines the essential information that can be collected for a thorough evaluation. Clinical assessment and history should be obtained from both the accused individual and any bed partners or witnesses. Key areas of inquiry include sleep patterns, prior history of parasomnias, medication and substance use and identifying potential triggers.

The investigational process may begin with a review of police records, including the charges and statements from victims and witnesses. A detailed interview of the client should be conducted, covering detailed psychiatric and medical histories, while also obtaining past relevant records. A thorough physical examination, along with complete biochemical and hematological/toxicological screening, is recommended. Neurophysiological evaluation may include electroencephalography (EEG) and brain imaging and PSG should be arranged to document sleep-related behaviors. Interviews with relatives and, when appropriate, obtaining the victim's account of the event are essential. Of significant importance is the victim's observation and perception of the perpetrator's level of awareness. The victim's medical and psychiatric history, along with any prior similar incidents or records, may help identify patterns of repeated accusatory behavior. Additional assessments may include phallometric testing to determine specifically if there is an erotic preference for children and or sexual violence, blood and/or urine screening for drug use and comprehensive psychological testing, with emphasis on personality assessment and psycho-diagnostics across all DSM-5 TR axes (American Psychiatric Association, 2022).

No forensic assessment is complete without an assessment of malingering. Malingering in a case where sexsomnia is impugned requires an accurate evaluation of the account of the accused, the victim and of witnesses. Individuals are knowledgeable about sexsomnia and the history provided can be fabricated at many levels. It can start with reporting a history of the problem, lack of awareness of the event alleged, having a current bed partner support the diagnosis by also being deceived by claims of being aware of the sexual act to outright lying by the accused. Having other partners verify that the problem existed previously may be of greater value. Noting that if the victim is a child of an ex-partner, there may be problems or reliability of the mother supporting sexsomnia if there is a perception that the child has been abused (R. v. Towgood, 2007; R. v. Maidment, 1984). Often sexsomnia is not demonstrated in a laboratory setting as are sleep walking episodes during a PSG recording (R. v. Luedecke, 2008).

We had a patient with Down syndrome and his wife sleeping in the clinic and he showed “amarous intent” while asleep. Evaluation of patterns of responding in psychological testing *L* (Lie), *F* (Infrequency), *K* (Defensiveness) scales on Minnesota Multiphasic Personality Inventory (MMPI), responding patterns on the Personality Assessment Inventory (PAI), the Paulus Deception Scale and the Structured Interview of Reported Symptoms (SIRS) and other rating scales of general malingering may be helpful but one should be alert that many of these assessment instruments do not or are not specifically designed to assess sexsomnia (Archer et al., 2001; Sellbom et al., 2010). Likewise, diagnosis of antisocial personality disorder or psychopathy can raise the index of suspicion of malingering. Clinicians must also remain vigilant against bias, given that a diagnosis of personality disorder by itself may not reliably differentiate malingerers from honest responders (Svete et al., 2025). Seizure disorders and dissociative disorders need to be excluded and can be difficult (Lanfranco et al., 2023). The generation of a differential diagnosis and careful consideration to rule out malingering is a must. Finally, seeking a second opinion may provide additional objective confirmation and support the overall assessment.

Furthermore, clinicians should familiarize themselves with the law as it pertains to automatism, particularly in cases where sexsomnia is alleged and understand whether it is likely to be treated as a mental disorder or as conduct giving rise to a full acquittal. These issues can be discussed with the Crown, defense counsel or the individual referring the accused. Clinicians should not hesitate to request relevant case law, as the application of legal principles to sleep-related behaviors, sexsomnia and automatism may evolve over time.

Lastly, clinicians are advised to inquire whether there are any additional undisclosed materials that should be considered. Information deemed irrelevant by legal counsel may prove invaluable to a sleep expert or forensic psychiatrist in substantiating or refuting a diagnosis of sexsomnia. Clinicians must also remain mindful that, regardless of who retains them, expert opinions provided to the court are expected to be impartial and directed to assisting the court.

### 2.16 Risk and safety assessment

a. Has the patient ever:

i. Left the bedroom/house during episodes?
ii. Attempted sexual acts with someone unable to consent?
iii. Had episodes in public places?
iv. Injured themselves or a partner?

b. Are there children or vulnerable individuals in the home?

When bed partners report sexsomnia within an otherwise healthy relationship, both individuals require education regarding the condition. It should be emphasized that consent cannot be given for egregious harm and that consent provided prior to falling asleep does not absolve responsibility for sexual acts committed against a sleeping partner. These principles should be explicitly discussed with the individual experiencing the disorder.

To date, reported legal cases have involved mostly men (Riha et al., 2023). However, it remains uncertain how courts would interpret a case involving a woman with sexsomnia engaging in sexual activity with another individual, regardless of whether the partner was unaware of the condition or had prior knowledge of it. This uncertainty must be evaluated against the foundational principle that consent must be affirmative. In many couples, there is an acceptance that sexual interaction may occur without consent and when one of the partners (usually male) is asleep.

### 2.17 Sleep study and previous evaluations

a. Any prior PSG sleep studies?
b. Any prior EEG studies?
c. Prior diagnoses of parasomnias or epilepsy?
d. Any documentation from previous clinicians?

Proper risk assessment for sexsomnia requires a thorough review of an individual's prior medical and sleep history. Investigators should examine any previous PSG or EEG studies, as these can provide objective evidence of sleep architecture abnormalities or epileptiform activity. Documentation of prior diagnoses of parasomnias or epilepsy is also critical, as these conditions may predispose to sexsomnia (Dubessy et al., 2017; Toscanini et al., 2021). Clinical notes or reports from previous healthcare providers can offer valuable context regarding symptom onset, frequency and triggers, facilitating a comprehensive evaluation of risk factors. Finally, two consecutive current PSG studies, a mean sleep latency test (MSLT) and a maintenance of wakefulness test (MWT) could aid in the diagnosis of sleep disorders and the assessment of sleepiness and alertness of the accused. Two consecutive PSG studies are recommended to mitigate the first-night effect (Byun et al., 2019).

### 2.18 Lifestyle factors

a. Work schedule (e.g., shift work)^°.*l*.^
b. Exercise patterns
c. Stress levels^°.*l*.^
d. Travel or jet lag

Lifestyle factors can significantly influence sleep continuity and increase the likelihood of arousals from deep sleep, potentially precipitating episodes of sexsomnia in susceptible individuals (Kurt Gök et al., 2021). Irregular work schedules, particularly shift work, disrupt circadian rhythms and sleep homeostasis (Kurt Gök et al., 2021). Exercise patterns, especially vigorous activity close to bedtime, can elevate physiological arousal and interfere with SWS. Elevated stress levels are associated with increased nocturnal awakenings and fragmented sleep, while travel across time zones or jet lag can further destabilize circadian timing (Willoughby et al., 2025). Collectively, these lifestyle-related factors create conditions that may trigger partial arousals during deep sleep, increasing vulnerability to sexsomnia.

### 2.19 Legal and forensic considerations

a. Any accusations of inappropriate sexual behavior?
b. History of legal issues arising from sleep episodes.
c. Documentation from previous legal reports/incidences.

While many of the forensic aspects of sleep are addressed in the body of this paper, this section addresses what needs to be focused on in a case of sleep-related sexual behaviors.

From a criminal law perspective, assaultive and sexual behaviors are closely linked where it is established that consent was not given. For any viable defense, it must be shown that the impugned sexual behaviors were committed in a state in which the individual lacked voluntary control. Such conduct would fall within the legal doctrine of automatism.

Two major issues arise. First, if the behaviors are likely to recur, a court may be inclined to characterize the conduct as a mental disorder automatism. However, in cases involving other sleep-related behaviors, such as sleep talking, singing or even sleep-driving, courts often attempt to impose parameters aimed at preventing recurrence rather than concluding that the individual was unaware of committing a criminal act. Conversely, if the episode is isolated, establishing a diagnosis of sexsomnia may be more challenging. If that diagnostic threshold is nonetheless met, it may be argued, consistent with R v Parks (R. v. Parks, 1992), that sexsomnia does not constitute a mental disorder and should instead be classified as a non-mental disorder automatism. In either scenario, the party raising the defense bears the burden of establishing automatism on a balance of probabilities. Establishing sexsomnia or other sleep-related sexual behaviors not under voluntary control is challenging in sexual assault cases, as the relevant evidence must originate from sources external to the accused. This often requires corroboration from accounts provided by the complainant, who may not always perceive themselves as a victim.

When this occurs, the credibility of collateral sources often becomes a significant issue. Although a current bed partner may support the diagnosis, such support raises concerns about potential bias and caution is warranted when relying on testimony from a spouse or current partner. Former partners may have fewer vested interests in supporting a claim of sexsomnia, particularly if the relationship ended poorly. Conversely, as illustrated in a recent case, a former partner may have observed relevant behaviors. However, if the alleged victim is a child of the former partner, they may be unwilling to support a sexsomnia claim for a variety of questionable reasons (R. v. Maidment, 1984).

The victim's account must be carefully evaluated, particularly when the events leading up to the alleged offense are contradicted by the accused. In such circumstances, reviewing transcripts of the victim's testimony from a preliminary hearing or trial may be especially helpful. In Maidment (R. v. Maidment, 1984), such contradictory accounts were central to the case.

In R. v. Towgood (2007), a prior sexual act with a child was deemed to have occurred during sexsomnia and the criminal offense that resulted was disposed of without a conviction. However, when the same act occurred again with another child and lab testing also indicated a problem of pedophilia, the sexsomnia defense was not upheld. In a complicated case such as this, both sexsomnia and pedophilia can coexist and in a state of sleep, if a child is present in the room, the person could act out sexually with the victim. When the perpetrator alleged that the child got into his bed, it raises different concerns.

After the first charge, detailed counseling about preventive measures needs to be taken to protect other people in the vicinity. There should be an appropriate treatment process for individuals with sexsomnia. This raises concerns about the legal liability of treating clinicians when advising the accused about the risk of recurrence and the measures needed to protect others sleeping in the vicinity. A concerning example involves a family camping together in a tent shared by six people. The sleeping arrangements place the father between the children, with the mother positioned at the foot of the tent. Two weeks after the trip, one daughter reports inappropriate contact by the father during a night in the tent, prompting understandable outrage. This father should be promptly diagnosed with collateral history from his wife and appropriately treated. At present, to our knowledge, no civil cases have been reported that involve clinician liability in cases of sexsomnia.

One of the authors (JG) was asked to provide an opinion regarding the presence of sexsomnia in an alleged victim. The relevance and admissibility of this type of testimony were vigorously challenged in court, particularly with respect to whether an expert may opine on the condition in an alleged victim. The question of whether expert evidence on sexsomnia in this context is admissible remains to be determined by the court.

Caution is warranted when experts are called to testify on such matters, and the admissibility of their evidence is governed by the principles set out in (R. v. Mohan, 1994). We await the court's decision on whether this testimony will ultimately be admitted.

## Appendices

**Table A1:** Investigation process of a case of sexual activity when the individual claims to be asleep.

1	Review the police records of the charges and victim/witness statements.
2	Interview the client taking an exhaustive psychiatric and medical history.
3	Arrange for a thorough physical examination along with a full biochemical and hematological screen.
4	Obtain past medical and psychiatric records.
5	Arrange for an electroencephalogram and brain scanning.
6	Interview relatives of the client.
7	Obtaining the victim's account of the event or interviewing the victim is important.
8	Arrange for polysomnography (two nights to overcome the first night effect).
9	Consider phallometric (erotic preference) testing.
10	Consider a blood or urine screen for drug abuse.
11	Psychological testing with a special emphasis on personality testing.
12	Psychodiagnostics looking at all DSM-V-TR axes.
13	Evaluate for malingering, antisocial personality disorder and psychopathy.
14	Generate a differential diagnosis and rule out malingering.
15	Consider a second opinion from a person who is not going to be considered biased or in a conflict.
16	Consider use of standard questionnaires (ESS, STOP-BANG, etc)

DSM-5 RT, diagnostic statistical manual of mental disorder, fifth edition text revision; ESS, epworth sleepiness scale.

## References

[B1] American Academy of Sleep Medicine (2023). International Classification of Sleep Disorders, 3rd Edn. Darien, IL: American Academy of Sleep Medicine.

[B2] American Psychiatric Association (2022). Diagnostic and Statistical Manual of Mental Disorders 5th Edn. text rev. Washington, DC: American Psychiatric Association.

[B3] Arab A. Rafie N. Amani R. Shirani F. (2023). The role of magnesium in sleep health: a systematic review of available literature. Biol. Trace Elem. Res. 201, 121–128. doi: 10.1007/s12011-022-03162-1 35184264

[B4] Archer R. P. Handel R. W. Greene R. L. Baer R. A. Elkins D. E. (2001). An evaluation of the usefulness of the MMPI-2 F(p) scale. J. Pers. Assess. 76, 282–295. doi: 10.1207/S15327752JPA7602_10 11393461

[B5] Battiato A. Dodet P. Chaumereuil C. Maranci J. B. Leu-Semenescu S. Gales A. Z. . (2025). Familial and sporadic non-rapid eye movement parasomnia in adults: clinical and sleep differences. Sleep 48:zsaf103. doi: 10.1093/sleep/zsaf103 40434875

[B6] Bornemann M. A. Mahowald M. W. Schenck C. H. (2006). Parasomnias: clinical features and forensic implications. Chest 130, 605–610. doi: 10.1378/chest.130.2.605 16899867

[B7] Byun J. H. Kim K. T. Moon H. J. Motamedi G. K. Cho Y. W. (2019). The first night effect during polysomnography, and patients' estimates of sleep quality. Psychiatry Res. 274, 27–29. doi: 10.1016/j.psychres.2019.02.011 30776709

[B8] Cankardas S. Schenck C. H. (2020). Sexual behaviors and sexual health of sexsomnia individuals aged 18–58. Int. J. Sex. Health 33, 29–39. doi: 10.1080/19317611.2020.1850597 38596470 PMC10807804

[B9] Cartwright R. D. (2014). Alcohol and NREM parasomnias: evidence versus opinions in the international classification of sleep disorders, 3rd edition. J. Clin. Sleep Med. 10, 1039–1040. doi: 10.5664/jcsm.4050 25221449 PMC4153104

[B10] Cramer Bornemann M. A. Schenck C. H. Mahowald M. W. (2019). A review of sleep-related violence: the demographics of sleep forensics referrals to a single center. Chest 155, 1059–1066. doi: 10.1016/j.chest.2018.11.010 30472024

[B11] Dubessy A. L. Leu-Semenescu S. Attali V. Maranci J. B. Arnulf I. (2017). Sexsomnia: a specialized non-REM parasomnia? *Sleep* 40. doi: 10.1093/sleep/zsw043 28364495

[B12] Fietze I. Barthe C. Hölzl M. Glos M. Zimmermann S. Bauer-Diefenbach R. . (2016). The effect of room acoustics on the sleep quality of healthy sleepers. Noise Heal. 18, 240–246. doi: 10.4103/1463-1741.192480 PMC5187651 27762252

[B13] Guilleminault C. Moscovitch A. Yuen K. Poyares D. (2002). Atypical sexual behavior during sleep. Psychosom. Med. 64, 328–336. doi: 10.1097/00006842-200203000-00017 11914450

[B14] Harding E. C. Franks N. P. Wisden W. (2019). The temperature dependence of sleep. Front. Neurosci. 13:336. doi: 10.3389/fnins.2019.00336 31105512 PMC6491889

[B15] Holoyda B. J. Sorrentino R. M. Mohebbi A. Fernando A. T. Friedman S. H. (2021). Forensic evaluation of sexsomnia. J. Am. Acad. Psych. Law 49, 202–210. doi: 10.29158/JAAPL.200077-20 33579735

[B16] Howell M. J. (2012). Parasomnias: an updated review. Neurotherapeutics 9, 753–775. doi: 10.1007/s13311-012-0143-8 22965264 PMC3480572

[B17] Hu S. Chen Y. Chen J. Guo Y. Li Y. Shao Y. . (2024). The insensitivity of sleep to an unfamiliar sleeping environment in patients with insomnia disorder. Sleep Breath. 28, 467–473. doi: 10.1007/s11325-023-02914-0 37747601

[B18] Ingravallo F. Poli F. Gilmore E. V. Pizza F. Vignatelli L. Schenck C. H. . (2014). Sleep-related violence and sexual behavior in sleep: a systematic review of medical-legal case reports. J. Clin. Sleep Med. 10, 927–935. doi: 10.5664/jcsm.3976 25126042 PMC4106950

[B19] Iranzo A. (2018). Parasomnias and sleep-related movement disorders in older adults. Sleep Med. Clin. 13, 51–61. doi: 10.1016/j.jsmc.2017.09.005 29412983

[B20] Jiménez-Correa U. Santana-Miranda R. Barrera-Medina A. Martínez-Núñez J. M. Marín-Agudelo H. A. Poblano A. . (2022). Parasomnias in patients with addictions-a systematic review. CNS Spectr. 27, 58–65. doi: 10.1017/S1092852920001911 33092679

[B21] Kierlin L. Littner M. R. (2011). Parasomnias and antidepressant therapy: a review of the literature. Front. Psych. 2:71. doi: 10.3389/fpsyt.2011.00071 PMC3235766 22180745

[B22] Klein L. A. Houlihan D. (2010). Relationship satisfaction, sexual satisfaction, and sexual problems in sexsomnia. Int. J. Sex. Heal. 22, 84–90. doi: 10.1080/19317610903510489

[B23] Korkutata A. Korkutata M. Lazarus M. (2025). The impact of exercise on sleep and sleep disorders. npj Biol. Time Sleep 2, 1–10. doi: 10.1038/s44323-024-00018-w PMC12912324 41775863

[B24] Kurt Gök D. Ünal I. Aslan-Kara K. (2021). Evaluation of the effects of shift work on parasomnia prevalence. Chronobiol. Int. 38, 1500–1506. doi: 10.1080/07420528.2021.1932996 34107833

[B25] Lanfranco R. C. Martínez-Aguayo J. C. Arancibia M. (2023). Assessing malingering and personality styles in dissociative identity disorder: a case study. Neurocase 29, 141–150. doi: 10.1080/13554794.2024.2348218 38704614

[B26] Mahowald M. W. Schenck C. H. (2005). Insights from studying human sleep disorders. Nature 437, 1279–1285. doi: 10.1038/nature04287 16251953

[B27] Mainieri G. Loddo G. Provini F. (2021). Disorders of arousal: a chronobiological perspective. Clocks Sleep 3, 53–65. doi: 10.3390/clockssleep3010004 33494408 PMC7838780

[B28] Mangan M. A. (2004). A phenomenology of problematic sexual behavior occurring in sleep. *Arch*. Sex. Behav. 33, 287–293. doi: 10.1023/B:ASEB.0000026628.95803.98 15129047

[B29] Manni R. Terzaghi M. (2010). Comorbidity between epilepsy and sleep disorders. *Epilepsy Res*. 90, 171–177. doi: 10.1016/j.eplepsyres.2010.05.006 20570109

[B30] Munro N. A. (2020). Alcohol and parasomnias: the statistical evaluation of the parasomnia defense in sexual assault, where alcohol is involved. J. Forensic Sci. 65, 1235–1241. doi: 10.1111/1556-4029.14322 32259289

[B31] Ollila H. M. Sinnott-Armstrong N. Kantojärvi K. Broberg M. Palviainen T. Jones S. . (2024). Nightmares share genetic risk factors with sleep and psychiatric traits. Transl. Psych. 14:123. doi: 10.1038/s41398-023-02637-6 PMC10899618 38413574

[B32] Pallesen S. Saxvig I. W. Waage S. Schenck C. H. Bjorvatn B. (2025). The prevalence of sexsomnia in a general population sample. Arch. Sex. Behav. 54, 3495–3502. doi: 10.1007/s10508-025-03235-x 41044291 PMC12675602

[B33] Pressman M. R. (2007). Disorders of arousal from sleep and violent behavior: the role of physical contact and proximity. Sleep 30, 1039–1047. doi: 10.1093/sleep/30.8.1039 17702274 PMC1978391

[B34] Rauch L. Schneider T. Wendt C. (2024). Seeking professional help for sleep-related complaints. Front. Public Heal. 12:1430574. doi: 10.3389/fpubh.2024.1430574 PMC11655339 39703480

[B35] Riha R. L. Dodds S. Kotoulas S. C. Morrison I. (2023). A case-control study of sexualised behaviour in sleep: a strong association with psychiatric comorbidity and relationship difficulties. Sleep Med. 103, 33–40. doi: 10.1016/j.sleep.2023.01.019 36746108

[B36] R. v. Luedecke (2008). ONCA 716, 93 O.R. (3d) 89, 236 C.C.C. (3d) 317.

[B37] R. v. Maidment (1984). 10 CCC (3d) 512 (NSCA).

[B38] R. v. Mohan (1994). 2 SCR 9. doi: 10.25071/6v954298

[B39] R. v. Parks (1992). 2 SCR 871.

[B40] R. v. Towgood (2007). 74 WCB (2d) 657 (BCSC).

[B41] Sabé M. Sulstarova A. Glangetas A. De Pieri M. Mallet L. Curtis L. . (2025). Reconsidering evidence for psychedelic-induced psychosis: an overview of reviews, a systematic review, and meta-analysis of human studies. Mol. Psych. 30, 1223–1255. doi: 10.1038/s41380-024-02800-5 PMC11835720 39592825

[B42] Schenck C. H. Arnulf I. Mahowald M. W. (2007). Sleep and sex: what can go wrong? A review of the literature on sleep-related disorders and abnormal sexual behaviors and experiences. Sleep 30, 683–702. doi: 10.1093/sleep/30.6.683 17580590 PMC1978350

[B43] Schlarb A. A. Stuck B. A. Maurer J. T. . (2021). “Sleep disorders in children,” in Practice of Sleep Medicine: Sleep Disorders in Children and Adults, eds. B. A. Stuck, J. T. Maurer, A. A. Schlarb, et al. (New York: Springer Nature), 242–249.

[B44] Sellbom M. Toomey J. A. Wygant D. B. Kucharski L. T. Duncan S. (2010). Utility of the MMPI-2-RF (restructured form) validity scales in detecting malingering in a criminal forensic setting: a known-groups design. Psychol. Assess. 22, 22–31. doi: 10.1037/a0018222 20230148

[B45] Shapiro C. M. Trajanovic N. N. Fedoroff J. P. (2003). Sexsomnia–a new parasomnia? Can. J. Psych. 48, 311–317. doi: 10.1177/070674370304800506 12866336

[B46] Sprecher K. E. Ferrarelli F. Benca R. M. (2015). Sleep and plasticity in schizophrenia. Curr. Top. Behav. Neurosci. 25, 433–458. doi: 10.1007/7854_2014_366 25608723 PMC5117633

[B47] Stallman H. M. Kohler M. (2016). Prevalence of sleepwalking: a systematic review and meta-analysis. PLoS One 11:e0164769. doi: 10.1371/journal.pone.0164769 27832078 PMC5104520

[B48] Svete L. J. Tindell W. W. McLouth C. J. Allen T. S. (2025). A retrospective analysis of rates of malingering in a forensic psychiatry practice. J. Am. Acad. Psychiatry Law. 53, 1–8. doi: 10.29158/JAAPL.240083-24

[B49] Toscanini A. C. Marques J. H. Hasan R. Schenck C. H. (2021). Sexsomnia: case based classification and discussion of psychosocial implications. Sleep Sci. 14, 175–180. doi: 10.5935/1984-0063.20200057 34381582 PMC8340885

[B50] Trajanovic N. N. Mangan M. Shapiro C. M. (2007). Sexual behaviour in sleep: an internet survey. Soc. Psychiatry Psychiatr. Epidemiol. 42, 1024–1031. doi: 10.1007/s00127-007-0258-0 17932612

[B51] Willoughby A. R. Vallat R. Ong J. L. Chee M. W. L. (2025). Insights about travel-related sleep disruption from 1.5 million nights of data. Sleep 48:zsaf077. doi: 10.1093/sleep/zsaf077 40127035 PMC12246376

[B52] Zadra A. Pilon M. Montplaisir J. (2008). Polysomnographic diagnosis of sleepwalking: effects of sleep deprivation. Ann. Neurol. 63, 513–519. doi: 10.1002/ana.21339 18351640

